# Sustained Remission With Atezolizumab in a Frail, Geriatric Patient With Advanced-Stage Large Cell Neuroendocrine Carcinoma Lung

**DOI:** 10.1155/crom/2406678

**Published:** 2025-03-31

**Authors:** Mayank Kapoor, Praneet Bedi, Deepak Sundriyal, Ashita Jain, Ujjawal Shriwastav, Amit Sehrawat

**Affiliations:** ^1^Department of Medical Oncology Hematology, All India Institute of Medical Sciences, Rishikesh, India; ^2^Department of Medical Oncology, Max Superspeciality Hospital, Patparganj, New Delhi, India; ^3^Department of Pathology, All India Institute of Medical Sciences, Rishikesh, India; ^4^Department of Internal Medicine, Chitwan Medical College, Bharatpur, Nepal; ^5^Tribhuvan University, Kirtipur, Nepal

**Keywords:** advanced-stage cancer, atezolizumab, chemotherapy, geriatric patient, large cell neuroendocrine carcinoma

## Abstract

Large cell neuroendocrine carcinoma (LCNEC) is a rare, aggressive cancer primarily found in the lungs but can also occur in other organs. It is characterized by rapid progression and high metastatic potential. We present a case of advanced-stage LCNEC lung in a patient with a poor performance status (PS), requiring oxygen support. Imaging revealed a large right upper lobe mass, lymphadenopathy, with bronchial encasement and invasion into the superior vena cava, leading to SVC syndrome and pleural effusion. Biopsy and immunohistochemistry confirmed LCNEC. Due to the patient's poor PS, treatment began with low-dose single-agent chemotherapy (carboplatin), followed by etoposide and cisplatin after improvement. Local radiation was also administered, and the treatment plan was adjusted to include atezolizumab. After 10 cycles, the patient achieved complete remission, sustained for 6 years. This case highlights the complexities of managing advanced LCNEC in a geriatric patient and the effectiveness of a multidisciplinary approach and immunotherapy.

## 1. Introduction

Large cell neuroendocrine carcinoma (LCNEC) is a high-grade neuroendocrine carcinoma noted for its aggressive nature and poor overall prognosis. While commonly found in the lungs, LCNEC may also occur in other organs, such as the gastrointestinal tract. It accounts for around 3% of the lung cancer cases [1]. The World Health Organisation (WHO) classification of 2015 categorized neuroendocrine neoplasms of the lung into one group, subdivided into four types with distinct prognoses: typical carcinoid, atypical carcinoid (representing low and intermediate grade neuroendocrine tumors), small cell lung cancer (SCLC), and LCNEC (representing high-grade neuroendocrine carcinomas) [2]. LCNEC is characterized by large, poorly differentiated neuroendocrine cells with a high propensity for metastasis. Management typically involves a combination of surgery, chemotherapy, and radiation tailored to the patient's specific condition. This report presents a challenging case of advanced-stage LCNEC in a geriatric patient with poor performance status (PS), highlighting the effective use of a multidisciplinary approach and the significant role of immunotherapy in achieving long-term remission.

## 2. Case Presentation

A 67-year-old gentleman presented to the emergency department with right-sided chest and shoulder pain for 1 month's duration. It was accompanied by a nonproductive cough and no history of fever or weight loss. He had a chronic smoking history of 30 pack-years and was receiving tiotropium, formoterol, and budesonide metered-dose inhalers for chronic obstructive pulmonary disease over the past 4 years. On examination, he was frail, with an Eastern Cooperative Oncology Group (ECOG) PS of 4, tachypnea (respiratory rate of 36 breaths per minute), tachycardia (pulse rate of 110 beats per minute), oxygen saturation of 84%, and blood pressure of 112/84 mm hg in the upper limb. He exhibited a facial plethora and prominent thoracic veins, necessitating high-flow oxygen therapy. Examination of the respiratory system revealed decreased breath sounds over the right infraclavicular area, accompanied by stony dullness in the inferior axillary and infrascapular regions. The remainder of the systemic examination was unremarkable.

**Figure 1:**
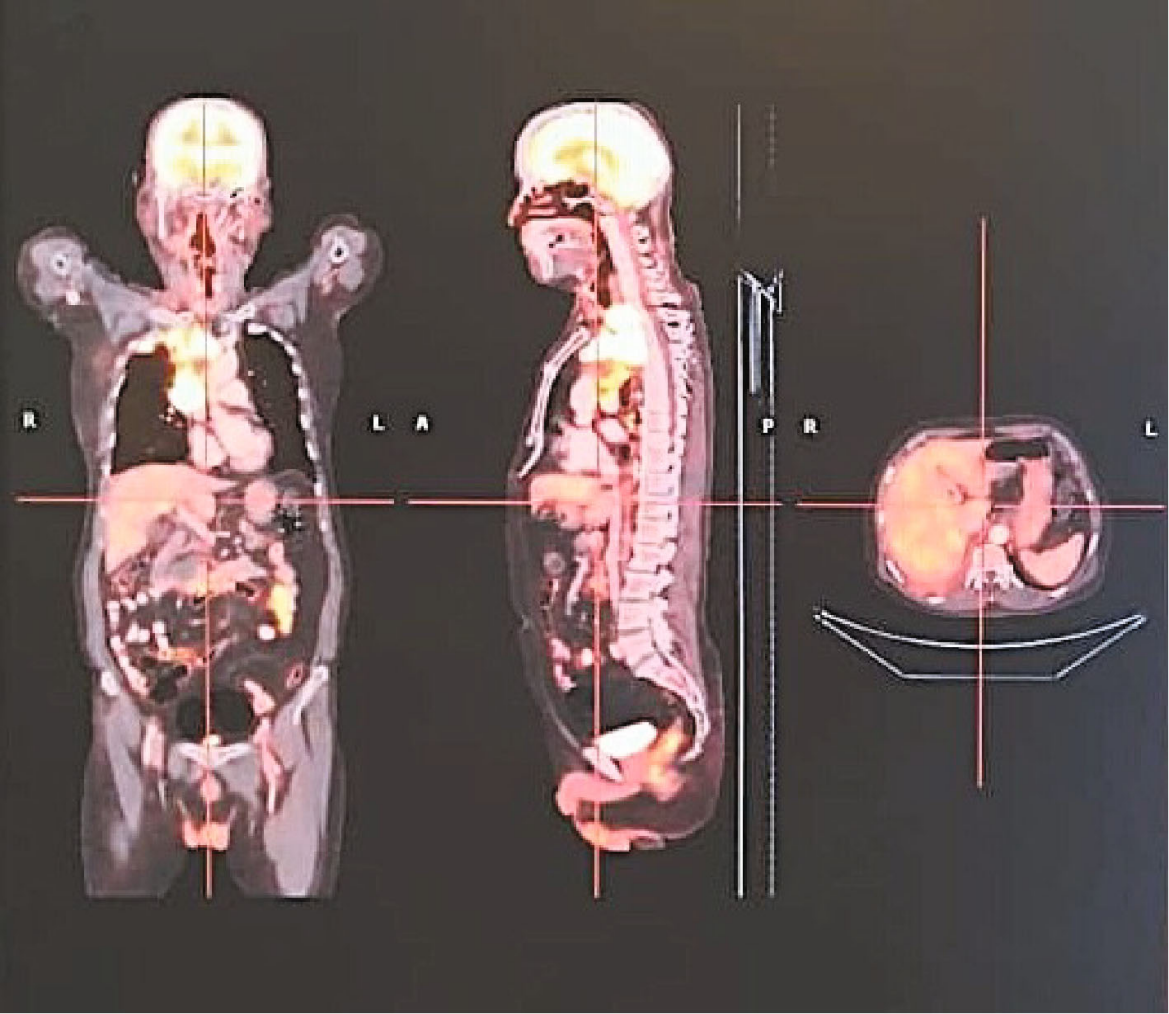
PET-CT showing a large mass in the right upper lobe of the lung, with associated mediastinal lymphadenopathy and right-sided pleural effusion.

**Figure 2:**
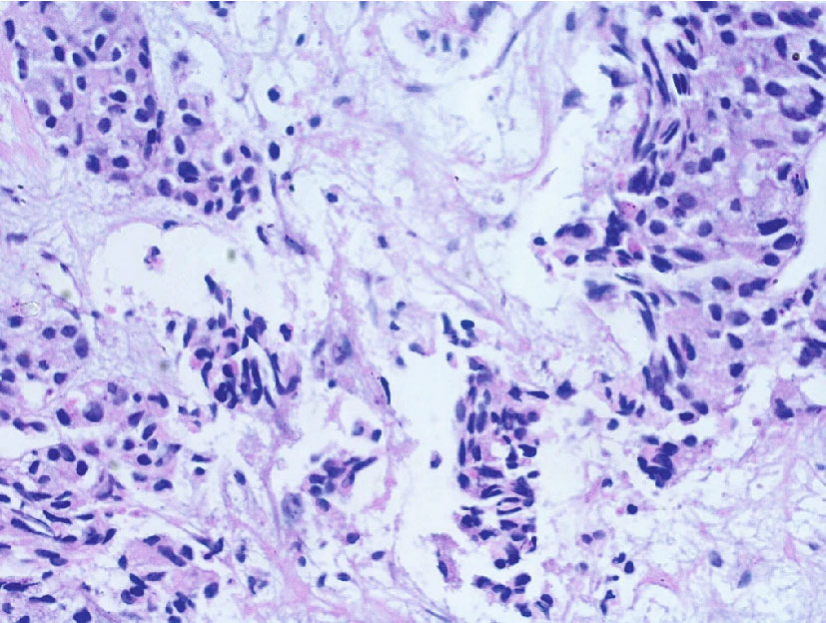
Photomicrograph of lung tissue with large atypical cells (hematoxylin & eosin; 40x).

**Figure 3:**
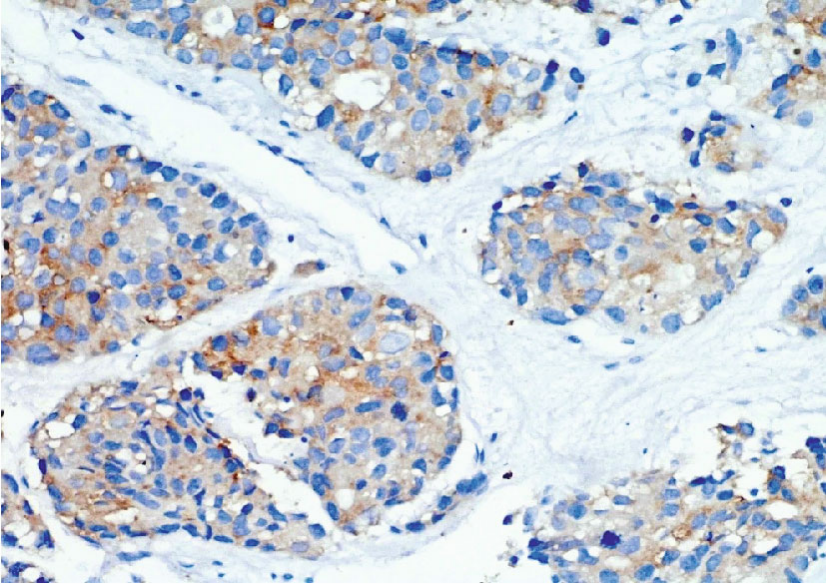
Positive expression of synaptophysin in large cell neuroendocrine carcinoma (40x).

Arterial blood gas analysis indicated respiratory acidosis, while routine biochemical investigations were within normal limits. Given the patient's shoulder pain, he underwent magnetic resonance imaging (MRI) of the cervical spine from a previous facility, revealing spondylotic and disc degenerative changes with multiple disc bulges and nerve root compression. Incidentally, MRI findings also demonstrated a mass lesion in the right upper lobe of the lung and right supraclavicular lymphadenopathy. Ultrasonography of the chest and abdomen showed right-sided pleural effusion. Subsequently, a positron emission tomography-computed tomography (PET-CT) scan revealed a large mass in the right upper lobe of the lung, with associated mediastinal lymphadenopathy and right-sided pleural effusion (Figure 1). Further diagnostic workup, including a CT venogram, indicated encasement of the right upper lobe bronchus, superior vena cava (SVC) invasion, and extension into the right internal jugular vein, leading to SVC syndrome. A lung mass biopsy confirmed the presence of large, poorly differentiated atypical cells. Immunohistochemistry (IHC) revealed focal positivity for cytokeratin (punctate dot-like) and CD56, with some tumor cells positive for synaptophysin and chromogranin. The tumor cells were negative for CK7, CK20, TTF-1 P63, CK5/6, napsin, and LCA. The Ki-67 proliferative index of the tumor was 85%–90%. Thus, a final diagnosis of high-grade neuroendocrine carcinoma, large cell type with SVC syndrome, clinical stage T4N1M1a was established (Figures 2 and 3).

**Figure 4:**
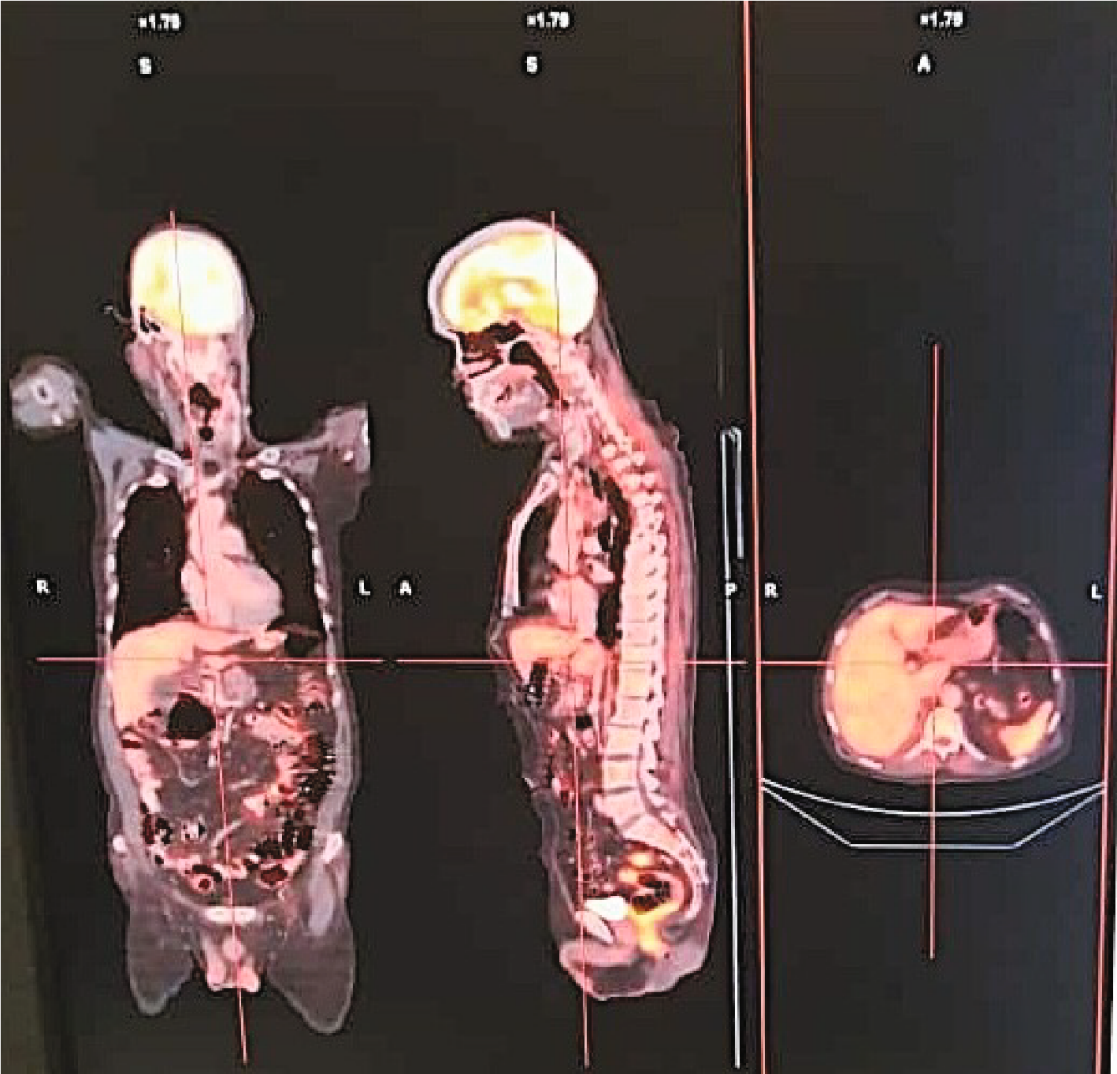
Imaging post three cycles revealing a partial response, with a decrease in size and avidity of the right lung mass and mediastinal lymphadenopathy.

Therapeutic pleurocentesis was performed in the emergency department to alleviate dyspnea, accompanied by oxygen therapy via a mask. The patient received nebulization with budesonide and bronchodilators. The family was counseled in detail regarding the advanced nature of the disease and its poor prognosis, leading to a shared decision to proceed with chemotherapy. Given the patient's poor ECOG-PS of 4 and a Cancer and Aging Research Group (CARG) chemotoxicity score of 8 (indicating a 54% risk of Grade 3 or higher toxicity), an initial treatment plan was established involving two cycles of reduced-dose (AUC 1.5) single-agent carboplatin administered weekly. Following 2 weeks of therapy, the patient demonstrated an improvement in PS to two, along with favorable arterial blood gas parameters. Signs and symptoms of SVC syndrome were no longer apparent. The treatment regimen was then adjusted to include a combination of etoposide (80 mg/m^2^ for 3 days) and cisplatin (75 mg/m^2^ on day one) every 3 weeks. Due to the advanced stage and complex nature of the disease, the treatment plan was revised to incorporate atezolizumab (a programmed cell death ligand 1 (PD-L1) inhibitor) at a dosage of 1200 mg once every 3 weeks. This decision was informed by emerging evidence regarding the efficacy of immunotherapy in SCLC and the patient's clinical improvement [3]. Imaging conducted after three cycles demonstrated a partial response characterized by decreased size and avidity of the right lung mass and mediastinal lymphadenopathy (Figure 4). Local radiation therapy (30Gy in 10#) followed chemotherapy after six cycles to address the local tumor burden, followed by prophylactic cranial irradiation (PCI) (25Gy in 10#). Although PCI has no definite survival advantage, it helps decrease the incidence of cerebral metastases, thus improving quality of life [4]. Chemotherapy was discontinued after six cycles, while atezolizumab was continued.

**Figure 5:**
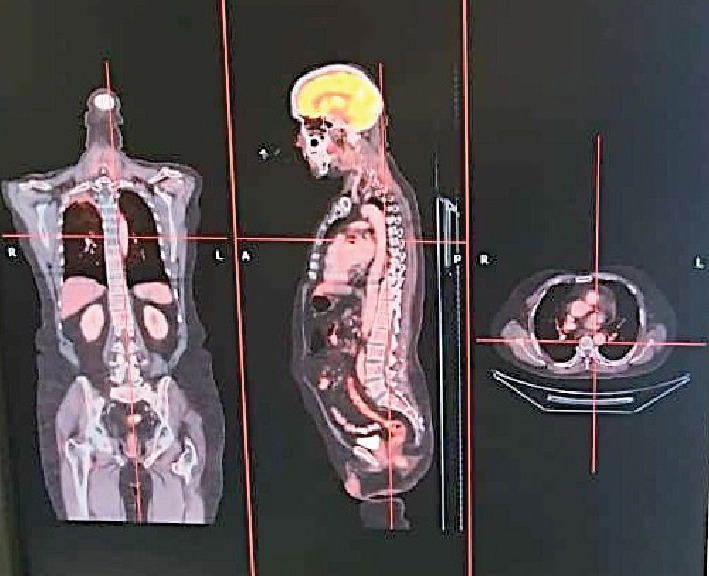
PET-CT showing complete response after 10 cycles of atezolizumab, (sustained for the past 6 years).

Atezolizumab was administered for a total duration of 2 years at a frequency of once every 3 weeks. The patient achieved complete remission after 10 cycles of atezolizumab, sustained for 6 years (Figure 5). Follow-up imaging consistently demonstrated no evidence of disease recurrence. The patient experienced minimal side effects (Grade 1 fatigue and nausea) and significantly improved PS and quality of life. There were no immune-related adverse events.

## 3. Discussion

LCNEC presents significant management challenges, especially in geriatric patients with advanced-stage disease and poor PS. In this case, the combined use of chemotherapy and immunotherapy proved effective in achieving and maintaining long-term remission. The initial treatment with carboplatin, followed by etoposide and cisplatin, was crucial in reducing the tumor burden, while atezolizumab played a key role in achieving complete remission. This case underscores the importance of a multidisciplinary approach and the potential for immunotherapy to offer substantial benefits even in difficult-to-treat cases. Recent advances in the field of immune checkpoint inhibitors (ICIs) have dramatically altered survival outcomes and management strategies for lung cancer. Atezolizumab and durvalumab (PD-L1 inhibitors) have shown increased overall survival (OS) rates in patients with extensive-stage SCLC in combination with first-line chemotherapy [3, 5]. Many studies suggest the application of SCLC-based regimens in LCNEC. The median OS for SCLC regimens compared to non-small cell lung cancer (NSCLC) regimens was reported as 16.5 months versus 9.2 months [6]. However, only a limited number of studies have evaluated the role of ICIs in LCNEC. Improvement in OS from approximately 8 months to 12 months following the addition of atezolizumab to etoposide and a platinum doublet was observed (*p* = 0.20, 95% CI 0.06–0.79) in a nonrandomized study involving 17 patients, as compared to the platinum doublet alone. The median progression-free survival (PFS) in the combination arm was 6.6 months versus 6.3 months in the platinum doublet arm (*p* = 0.83, 95% CI 0.97–1.15), which was statistically nonsignificant [7]. Patients given ICIs demonstrated 12- and 18-month survival rates of 34% and 29%, respectively, while the group not receiving ICIs exhibited 24% and 15% rates, respectively [8]. A recent Moffitt Cancer Center and Research Institute study suggested a potential role of combined immunotherapy with chemotherapy in LCNEC with a satisfactory safety profile [9]. Kadota et al. reported a complete response maintained for 4 years with second-line pembrolizumab therapy in a patient with advanced-stage LCNEC [10]. This case report illustrates the successful management of advanced LCNEC in a geriatric patient with poor PS through chemotherapy and immunotherapy. A recent report has indicated OS data extending up to 60 months with atezolizumab [11]. To date, this is the only report demonstrating a sustained response, reporting a 72-month OS, which continues despite the cessation of therapy. The sustained remission achieved with atezolizumab emphasizes the need for continued exploration of immunotherapy in the treatment of aggressive neuroendocrine tumors. It should be noted that aging leads to immune dysregulation, and hence, geriatric patients tend to have more immune-related adverse events, especially those with poorer PS [12]. A multidisciplinary approach remains essential in managing complex cancer cases, ensuring optimal outcomes, safety, and improved quality of life for patients.

## 4. Conclusion

• A shared decision with family members and the initial few sitting of a single agent, low-dose chemotherapy can effectively reduce tumor burden. This should be the initial approach followed by full-dose chemotherapy in geriatric, frail patients otherwise unfit for chemotherapy.
• With the integration of atezolizumab, sustained remission is possible in advanced LCNEC of the lung, challenging its traditionally poor prognosis and highlighting the need for further research.

## Data Availability

The data supporting this study's findings are available from the corresponding author upon reasonable request.
